# Diagnostic performance of preoperative ultrasound for traumatic brachial plexus root injury: A comparison study with an electrophysiology study

**DOI:** 10.3389/fneur.2022.1077830

**Published:** 2023-01-06

**Authors:** Ailin Liu, Xiaotian Jia, Li Zhang, Xiaoyun Huang, Weimin Chen, Lin Chen

**Affiliations:** ^1^Department of Medical Ultrasound, Huashan Hospital, Fudan University, Shanghai, China; ^2^Department of Hand Surgery, Huashan Hospital, Fudan University, Shanghai, China

**Keywords:** ultrasound, brachial plexus, trauma, root injury, electrophysiology, comparison

## Abstract

**Purpose:**

Accurate preoperative assessment for traumatic brachial plexus injury (BPI) is critical for clinicians to establish a treatment plan. The objective of this study was to investigate the diagnostic performance of preoperative ultrasound (US) through comparison with an electrophysiology study (EPS) in the assessment of traumatic brachial plexus (BP) root injury.

**Materials and methods:**

We performed a retrospective study in patients with traumatic BPI who had preoperative US and EPS, excluding obstetric palsy and other nontraumatic neuropathies. US examination was performed on an EPIQ 5 color Doppler equipment. EPS was performed on a Keypoint 9033A07 Electromyograph/Evoked Potentials Equipment, testing electromyography (EMG), nerve conduction studies (NCS), and somatosensory evoked potentials (SEP). Each BP root of all patients was assessed by US and EPS as completely injured or incompletely injured, respectively. Sensitivity, specificity, positive predictive value (PPV), negative predictive value (NPV), and accuracy were calculated based on the correlation with intraoperative findings. The accuracy of US and EPS were compared using the McNemar test. The added benefit of US was evaluated by comparing the sensitivity and specificity between the combined tests with EPS using the McNemar test.

**Results:**

This study included 49 patients with traumatic BPI who underwent BP surgeries from October 2018 to September 2022. Surgical exploration confirmed 89 completely injured BP roots in 28 patients. US correctly detected 80 completely injured BP roots (sensitivity, 0.899; specificity, 0.981; PPV, 0.964; NPV, 0.944; accuracy, 0.951). EPS correctly detected 75 completely injured BP roots (sensitivity, 0.843; specificity, 0.929; PPV, 0.872; NPV, 0.912; accuracy, 0.898). US showed significantly higher accuracy than EPS (*p* = 0.03). When combining US and EPS for completely injured BP root detection, the sensitivity of the inclusive combination (0.966) was significantly higher than EPS alone (*p* = 0.000977), and the specificity of the exclusive combination (1.000) was significantly higher than EPS alone (*p* = 0.000977).

**Conclusion:**

Preoperative US is an effective diagnostic tool in the assessment of traumatic BP root injury. US had higher accuracy than EPS in this study. Sensitivity and specificity were significantly higher than EPS when US was combined with EPS.

## 1. Introduction

Traumatic brachial plexus injury (BPI) commonly affects younger people and causes significant disability of the upper extremity. Early exploration and nerve repair or nerve grafting are recommended in open sharp injuries, while the management of closed injuries requires patience and careful monitoring (1–5). As nonsurgical treatment is likely to have a good prognosis, surgery is not recommended for some patients, such as those with neuropraxia (4–6). Therefore, accurate preoperative assessment to stratify patients who require surgical intervention by identifying a complete injury is critical for establishing a treatment plan.

Apart from clinical symptoms and physical examinations, an electrophysiology study (EPS) is one of the most commonly used methods for preoperative assessments of traumatic BPI (3, 7–10). However, in complete injuries, EPS cannot provide reliable results within 3 to 4 weeks (1, 3, 7–9, 11), which might decrease the possibility of early surgical intervention and better functional outcomes (12, 13). Currently, ultrasound (US) has gained its role as a noninvasive and cost-effective imaging modality through recent successful attempts to assess brachial plexus (BP) neuropathy, showing that US might be a valuable alternative method (14–24).

To the best of our knowledge, although there have been a series of publications that reported the sensitivities of US for BPI diagnosis, ranging from 0.75 to 0.80 (16–18, 20, 24–26), fewer studies reported the specificity, and the sample sizes were limited (18, 20, 24). Furthermore, there is a shortage of published studies comparing US and EPS in a head-to-head fashion in the same set of patients with BPI (27, 28). Therefore, the aim of this study was to investigate the diagnostic performance of US through comparison with EPS in the detection of completely injured BP roots.

## 2. Materials and methods

This retrospective study was approved by the ethical institutional review board of Huashan Hospital, and all procedures were performed according to the Helsinki Declaration. Patients with traumatic BPI who underwent BP surgeries between October 2018 and September 2022 at Huashan Hospital, Fudan University (Shanghai, China), were included in this study. We searched our database of patients who (1) had a clear history of trauma; (2) had suspected BP root injury based on physical examination; and (3) underwent US and EPS examination before BP surgery. The exclusion criteria were as follows: (1) patients with obstetric plexus palsy, (2) patients who underwent previous BP surgery, (3) patients who showed obvious functional recovery after nonsurgical treatment before surgery, and (4) patients with other nontraumatic neuropathies. Medical records of each patient, such as age, sex, etiology, treatment history, US, and EPS results, were reviewed.

**Figure 1:**
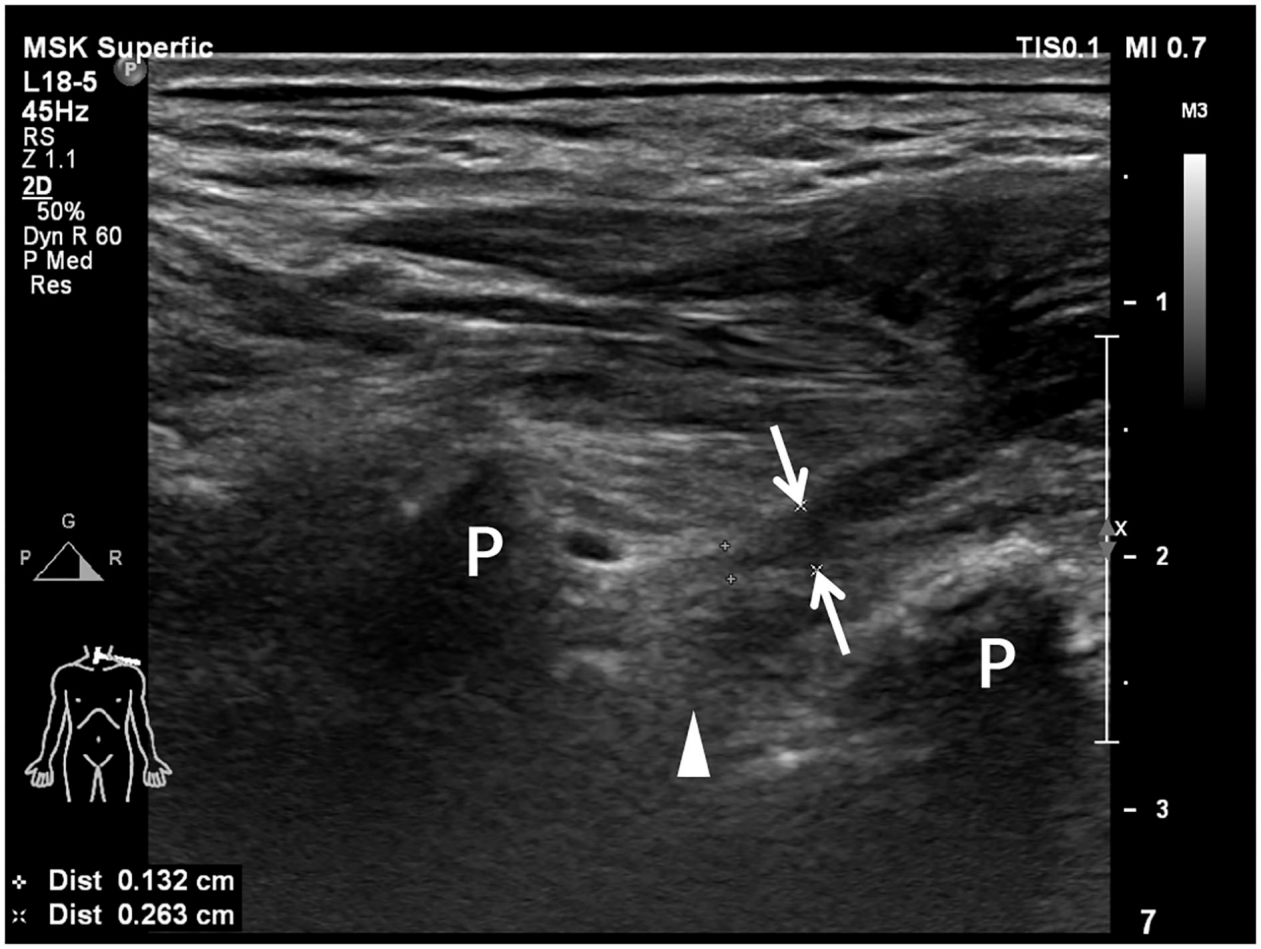
Longitudinal sonogram shows a hypoechoic irregular neural stump (arrows) with a blind-ending of the hypoechoic brachial plexus root. An empty neural foramen (arrowhead) is visualized, indicating preganglionic avulsion of the nerve root. Transverse processes (P) of cervical vertebrae are shown as hyperechoic bone prominences with posterior acoustic shadowing.

**Figure 2:**
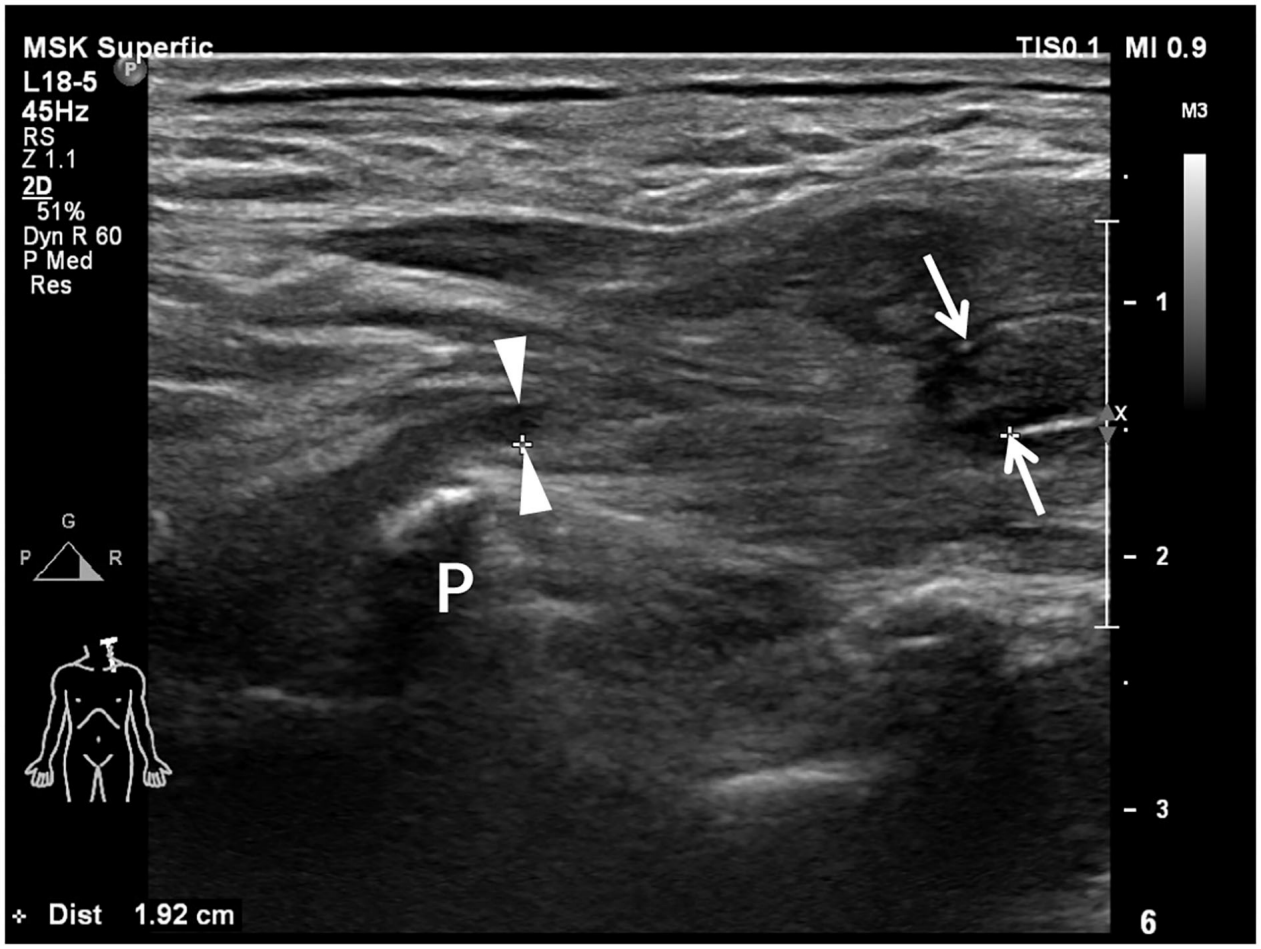
Longitudinal sonogram shows a completely transected brachial plexus root with a 1.92-cm-long neural gap between the proximal end (arrowheads) and the distal end (arrows). The proximal root end is adjacent to the transverse process (P). The caliber of the distal root end is enlarged in comparison with the proximal root end.

**Figure 3:**
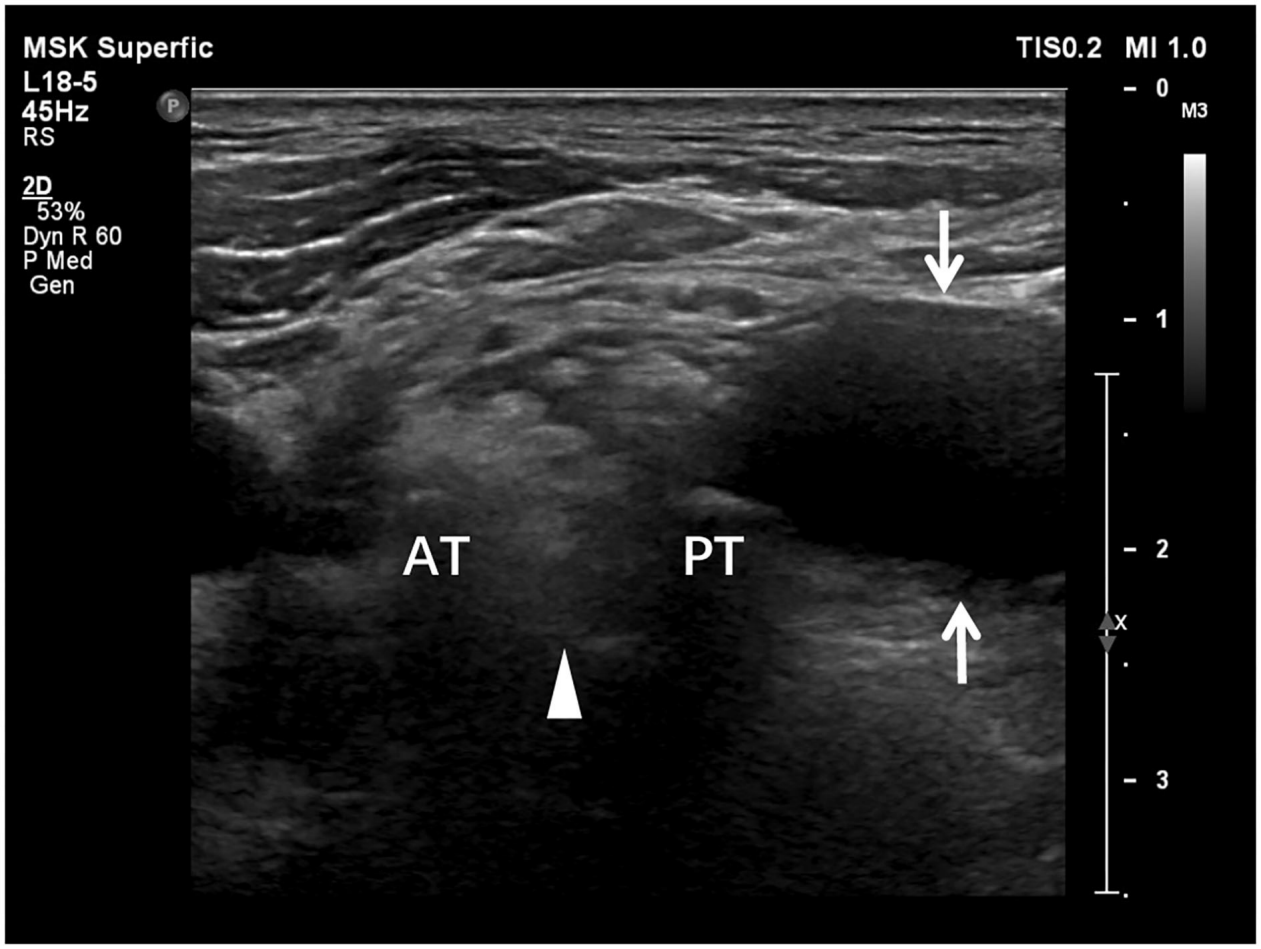
Transverse sonogram shows the accumulated cerebrospinal fluid, i.e., pseudomeningocele, adjacent to the intervertebral foramen. The cerebrospinal fluid (arrows) appears anechoic. The absence of brachial plexus root (arrowhead) between anterior (AT) and posterior tubercles (PT) represents preganglionic root avulsion.

**Figure 4:**
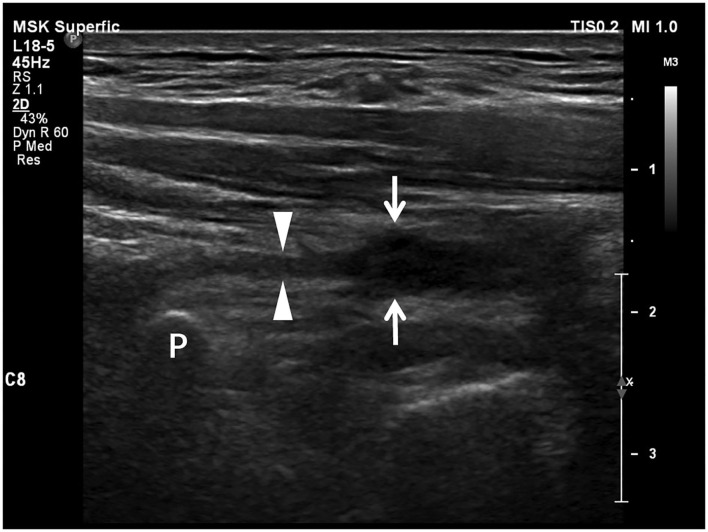
Longitudinal sonogram shows a neuroma-like enlargement of a brachial plexus root. The proximal part (arrowheads) of the nerve root adjacent to the transverse process (P) appears as a thin stripe. The distal part (arrows) of the root is hypoechoic and significantly enlarged as a neuroma losing smooth contour and regularly arranged fascicles due to retraction.

Preoperative US examination, including image collection and diagnosis, was performed by a certified radiologist with 9 years of experience in peripheral nerve imaging who was blinded to EPS and other imaging results. An EPIQ 5 color Doppler equipment (Philips Medical Systems, Bothell, WA, USA) was used for the standardized examination protocol. High-frequency linear-array transducers, namely, L18-5 MHz and L12-5 MHz, were used for deep structures. The patient was in a supine position with the neck extended to the noninjured side. As traditionally advised, US scanning started from the cranial part of the BP, e.g., C5 root, after identifying its corresponding transverse processes in a horizontal plane on the supraclavicular fossa. The C7 transverse process was the most commonly used bony landmark to locate the C7 root. Thus, the C5 to C8 roots could be depicted in the interscalene groove, arranged in a line as a series of hypoechoic nodules between the anterior and middle scalene muscles (29). The deepest T1 root could be visualized caudal to the C8 root coursing on top of the first rib. Each BP root was scanned along its inferior and lateral courses and tracked proximally in the transverse section and possible longitudinal section. In cases of variations, there might be BP roots coursing anterior to the anterior scalene muscle or piercing the muscle belly instead of traveling inside the interscalene groove; a rudimentary anterior tubercle might be visualized at the C7 transverse process; and a cervical rib might also appear (30). As such, following the caudal-to-cranial tracking technique by shifting the probe back and forth could effectively clarify the C5 to T1 BP roots and recognize variations (14, 30). Gentle tilting of the probe during the US scanning helped better delineate the edges of the nerve epineurium (31). The continuity of the BP root epineurium and fascicles and the existence of any accumulated cerebrospinal fluid or neuroma-like enlargement were recorded as observed (Supplementary Table 1). In US examination, a completely injured BP root was recorded as a positive result, which should have at least one of the following appearances: (1) neural stump (Figure 1), (2) neural gap (Figure 2), (3) cerebrospinal fluid adjacent to the intervertebral foramen (Figure 3), and (4) neuroma-like enlargement adjacent to the transverse process (Figure 4).

Preoperative EPS examination was performed by a certified neurophysiologist blinded to the US and other imaging results, following the information collection forms reflecting the content of examination standards (Supplementary Tables 2–5). A Keypoint 9033A07 Electromyograph/Evoked Potentials Equipment (Alpine bioMedApS, Skovlunde, DK-2740 Denmark) was used. The patients were asked to wait for at least 30 min before the examination, and room temperature (25°C) was required during the examination. Needle electromyography (EMG), nerve conduction studies (NCS), and somatosensory-evoked potential (SEP) were tested for each patient. Sensory NCS included sensory nerve conduction velocity (SNCV) and sensory nerve action potential (SNAP). Motor NCS included motor nerve conduction velocity (MNCV) and compound muscle action potential (CMAP). EMG was performed on relevant muscle sets using disposable concentric needle electrodes. Motor unit action potential (MUP) recruitment pattern characteristics were used to determine injury presence, severity, and recovery. Diagnosis of BPI was finally determined by the results of these EPS tests according to the anatomy of the brachial plexus. In EPS, a completely injured BP root was recorded as a positive result, which should meet all these characteristics: (1) abnormal spontaneous activities; (2) no motor units; and (3) no CMAP or MNCV.

The intraoperative findings were used as the reference standard. Exploratory BP surgeries were made *via* supraclavicular incisions. Observation and evaluation of the continuity of each BP root were performed up to the intervertebral foramen under a microscope. The sensitivity, specificity, positive predictive value (PPV), negative predictive value (NPV), and diagnostic accuracy of US and EPS for detecting completely injured roots were calculated using a 2 × 2 table. The statistical difference in diagnostic accuracy between US and EPS was examined by the exact, two-sided McNemar test using VassarStats.net (http://vassarstats.net/). To evaluate the added benefit of US when combined with EPS, sensitivity and specificity were calculated based on two models, namely, inclusive combination (if the result of either US or EPS was positive, it was considered positive) and exclusive combination (if both the results of US and EPS were positive, it was considered positive). Then, the sensitivity and specificity of these two models were compared with those of EPS alone by the exact, two-sided McNemar test. A *p*-value of 0.05 was considered the limit for a statistically significant difference.

## 3. Results

### 3.1. Patient characteristics

The clinical characteristics of the 49 patients are summarized in Table 1. Among them, 40 of the patients were men and nine were women. The majority of patients had a trauma of collision (41). The other patients suffered from stretch (4), crush (1), incision (1), and iatrogenic injuries (2). The age of the patients ranged from 5 to 78 years, with an average age of 42.6 years. The median time interval between injury and surgery was 87 days. The median time intervals between injury and preoperative examination were 75 days and 68 days for US and EPS, respectively.

**Table 1:** Demographic characteristics of 49 patients.

**Variables**	**Value**
**Sex**	
Men	40 (82%)
Women	9 (18%)
**Injury-involved side**	
Left	27 (55%)
Right	22 (45%)
**Etiology**	
Collision	41 (84%)
Stretch	4 (8%)
Crush	1 (2%)
Incision	1 (2%)
Iatrogenic injury	2 (4%)
**Age (years)**	Mean ± SD (range)
	42.6 ± 16.8 (5–78)
**Time interval (days)**	Median (Q1, Q3; Min–Max)
Between injury and surgery	87 (42, 137; 4–1,193)
Between injury and US examination	75 (32, 123; 0–1,189)
Between injury and EPS	68 (29, 123; 0–1,188)
Between US examination and surgery	3 (2, 6; 0–94)
Between EPS and surgery	6 (3, 12; 1–94)

### 3.2. Diagnostic performance of US and EPS

Surgical exploration confirmed 89 completely injured roots in 28 patients. US correctly detected 80 completely injured BP roots, with 80 true-positive results, 153 true-negative results, three false-positive results, and nine false-negative results (Table 2). EPS correctly detected 75 completely injured BP roots, with 75 true-positive results, 145 true-negative results, 11 false-positive results, and 14 false-negative results (Table 2). The number of positive and negative cases based on preoperative US and EPS examination is shown by root level in Table 2. In our study, US had fewer false-positive and false-negative results than EPS (Table 2). The overall sensitivity, specificity, PPV, NPV, and accuracy of detecting completely injured BP roots by US were 0.899, 0.981, 0.964, 0.944, and 0.951, respectively (Table 3). The overall sensitivity, specificity, PPV, NPV, and accuracy of detecting completely injured BP roots by EPS were 0.843, 0.929, 0.872, 0.912, and 0.898, respectively (Table 3). There was a significant difference in diagnostic accuracy between US and EPS (*p* = 0.03, Table 4).

**Table 2:** Preoperative US and EPS examination of completely Injured BP roots in correlation with intraoperative results.

**BP roots**	**Number of examined BP roots**	**Intraoperative results**		**US**				**EPS**			

		**Positive**	**Negative**	**True-positive**	**True-negative**	**False-positive**	**False-negative**	**True-positive**	**True-negative**	**False-positive**	**False-negative**
C5-T1	245	89	156	80	153	3	9	75	145	11	14
C5	49	19	30	17	29	1	2	17	28	2	2
C6	49	18	31	18	31	0	0	17	27	4	1
C7	49	21	28	19	28	0	2	16	26	2	5
C8	49	15	34	13	32	2	2	12	32	2	3
T1	49	16	33	13	33	0	3	13	32	1	3

**Table 3:** The sensitivity, specificity, PPV, NPV, and accuracy of preoperative US and EPS examination of completely injured BP roots.

**BP Roots**	**Number of examined BP roots**	**Number of completely-injured BP roots confirmed in surgery**	**Sensitivity of preoperative examination**		**Specificity of preoperative examination**		**Positive predictive value of preoperative examination**		**Negative predictive value of preoperative examination**		**Accuracy of preoperative examination**	

			**US**	**EPS**	**US**	**EPS**	**US**	**EPS**	**US**	**EPS**	**US**	**EPS**
C5-T1	245	89	0.899	0.843	0.981	0.929	0.964	0.872	0.944	0.912	0.951	0.898
C5	49	19	0.895	0.895	0.997	0.933	0.944	0.895	0.935	0.933	0.939	0.918
C6	49	18	1.000	0.944	1.000	0.871	1.000	0.910	1.000	0.964	1.000	0.898
C7	49	21	0.905	0.762	1.000	0.929	1.000	0.889	0.933	0.839	0.959	0.857
C8	49	15	0.867	0.941	0.941	0.941	0.867	0.857	0.941	0.914	0.918	0.898
T1	49	16	0.813	0.813	1.000	0.970	1.000	0.929	0.917	0.914	0.939	0.918

**Table 4:** Paired contingency table of diagnostic accuracy of preoperative US and EPS examination of completely injured BP roots.

	**EPS correct**	**EPS error**	**Total**
US correct	211	22	233
US error	9	3	12
Total	220	25	245

### 3.3. Combination of US and EPS

The sensitivity of the inclusive combination of US and EPS was significantly higher than EPS alone (*p* = 0.000977), while the difference in specificity was not statistically significant (*p* = 0.25, Table 5). The specificity of the exclusive combination of US and EPS was significantly higher (*p* = 0.000977), while the sensitivity was significantly lower (*p* = 0.03125) compared to EPS alone (Table 6).

**Table 5:** Comparison of sensitivity and specificity between EPS and the inclusive combination of US and EPS.

	**Inclusive combination (US or EPS)**	**EPS**	*p*-value^*^
Sensitivity	0.966	0.843	0.000977
Specificity	0.910	0.929	0.25

^*^Two-sided McNemar test.

**Table 6:** Comparison of sensitivity and specificity between EPS and the exclusive combination of US and EPS.

	**Exclusive combination (US and EPS)**	**EPS**	p-value^*^
Sensitivity	0.775	0.843	0.03125
Specificity	1.000	0.929	0.000977

^*^Two-sided McNemar test.

## 4. Discussion

A series of publications reported that both US and EPS are valuable tools for evaluating BPI (8, 25, 26, 32–35). However, to the best of our knowledge, published literature comparing the diagnostic performance of US and EPS on BPI is limited (27, 28). To investigate their diagnostic performance, US was compared head-to-head with EPS in 245 BP roots from 49 patients in our study by using intraoperative findings as the reference standard. Furthermore, the added benefit of US was shown when we combined it with EPS.

Our study showed a higher sensitivity (0.899) of US than previous reports (0.75–0.80) (16–18, 20, 24–26) and a higher accuracy of US (0.951) than EPS (0.898). One possible factor was that the radiologist in our study had extensive experience in peripheral nerve imaging. Another possible explanation was that the result of EPS might change during the spontaneous nerve recovery process, which might cause false diagnoses (3, 7–9, 11, 36).

The results obtained by US were consistent with EPS in 69 true-positive BP roots, 142 true-negative BP roots, and three false-negative BP roots. In this study, when US and EPS were both diagnosed positive, the BP roots were all completely injured. When US and EPS were both diagnosed negative, most (142/145) of the BP roots were not completely injured.

Despite the high concordance rate between US and EPS, 22 BP roots were correctly diagnosed by US but not by EPS, and 9 BP roots were correctly diagnosed by EPS but not by US. Notably, the inclusive combination of US and EPS could correct 11 out of 22 roots falsely diagnosed by EPS and 6 out of 9 roots falsely diagnosed by US. The exclusive combination of US and EPS could correct the other 11 out of 22 roots falsely diagnosed by EPS. Therefore, we suggest the inclusive and exclusive combination might be taken into consideration for assessing BP root injuries.

As a nerve conduction test, EPS could verify the continuity of a neural pathway to distinguish partial rupture from complete rupture when the neuroma-like enlargement is ambiguous for US. The advantages of Doppler US include high spatial resolution capabilities, portability, easy access, low cost, comparison with the contralateral side, and vascular information. Besides, this noninvasive and radiation-free method can generate real-time images, which allows extremity movement and muscle contraction during the scanning (37). A series of publications showed that US may provide useful morphological information (14–21, 23, 24, 36, 38) to help surgeons choose the appropriate surgical time and surgery type as well as refine surgical decisions in advance. Although the time for nerve exploration in closed injuries remains controversial, it is accepted that early nerve transfer for preganglionic root avulsion and early nerve repair or nerve grafts for postganglionic root rupture correlate with better outcomes (1, 4–6). Since the denervated muscle of the preganglionic avulsed root can only be reactivated by nerve transfer, it is vital to diagnose which roots were avulsed in preoperative preparation. If a preganglionic root avulsion is diagnosed, clinicians can better plan the nerve transfer surgery by choosing the suitable donor nerve and testing its function earlier. In addition to clarifying which roots are postganglionic ruptured, US can measure the distance between the neural stumps. An accurate measurement can help inform clinicians in advance to determine whether a neuroanastomosis can be performed or a nerve grafting surgery is necessary. US can visualize the internal texture and surrounding structures of the nerve (25, 31), which is a suitable method for noninvasive monitoring of lesions in continuity at the same anatomical site during spontaneous recovery (4–6). In addition, US can help in making a clinical decision about whether adopting neurolysis for treating continuous lesions with postganglionic swelling or compression when nonsurgical treatment does not lead to functional recovery after a period of time, usually at least 3–6 months based on the opinion of the majority of clinicians, is necessary (1, 4–6, 11–13). Moreover, the additional morphological information provided by US could improve the preoperational preparation of the surgery and, hence, reduce the time of surgery and the risk of infection.

There were three falsely diagnosed BP roots by both US and EPS. US images of these completely injured roots did not show any positive signs defined in this study, e.g., neural stump, neural gap, cerebrospinal fluid, or neuroma-like enlargement. In this study, two of these three roots (C8 and T1) were from one patient who was injured around 3 years before the examinations. Despite the operator-dependent nature (17–20, 23, 24), the prominent deficiency of US is its poor visualization of deep roots due to the anatomic location, edema, or obscuration by the bone (16–20, 23, 24, 36), which might be the reason for the relatively lower sensitivity of deeper roots in our study. Besides the features investigated in our study, ultrasonographic researchers also studied other parameters of histological structure that might support the diagnosis of peripheral nerve injuries. For example, in a recent publication, Ricci et al. recommended (semi)-quantitative measurement of the peripheral nerve internal structures and the nerve microcirculation by using high-frequency probes and high-sensitive color/power Doppler. The sonographic parameters of interest included (1) thickness of epifascicular epineurium; (2) texture of interfascicular epineurium; (3) number, size, and echogenicity of fascicles; and (4) intrafascicular/extrafascicular vascular signals. By combining these parameters, grayscale and vascular US allowed clear visualization of two pathological sonographic patterns. The first one was the hypoechioic nerve due to fascicular edema, which is usually observed in the acute phase of pathology. The second one was the hyperechoic nerve due to fibrotic involution of the interfascicular tissue and reduced number and size of fascicles, which correlated with the chronic phase of pathology. These abovementioned sonographic features could be considered potential additional sonographic features to augment the diagnostic accuracy of US in future studies, especially for neural stumps and neuroma-like enlargement (31).

The main bias of this study resulted from the observer of the reference standard, who also made the decision to operate and was not blinded to preoperative US or EPS results. Second, all the patients included in this retrospective study were surgical cases, which indicates that this study was enriched with severe patients with BPI who needed surgeries. This did not reflect the proportion of severe BPI in the real-world BPI population, so the performance of US or the combination of US and EPS needed to be further investigated in a medium or minor level of BPI.

## Data availability statement

The original contributions presented in the study are included in the article/Supplementary material, further inquiries can be directed to the corresponding authors.

## Ethics statement

The studies involving human participants were reviewed and approved by the Ethical Institutional Review Board of Huashan Hospital. Written informed consent to participate in this study was provided by the participants' legal guardian/next of kin.

## Author contributions

AL and XJ collected and analyzed the data and wrote the manuscript. AL, XJ, LZ, XH, WC, and LC collected the data. WC and LC supervised the analyzing and writing work. All authors contributed to the article and approved the submitted version.

## References

[1] Shin AY Spinner RJ Steinmann SP Bishop AT. Adult traumatic brachial plexus injuries. J Am Acad Orthop Surg. (2005) 13:382–96. 10.5435/00124635-200510000-00003 16224111

[2] Kato N Htut M Taggart M Carlstedt T Birch R. The effects of operative delay on the relief of neuropathic pain after injury to the brachial plexus: a review of 148 cases. J Bone Joint Surg. (2006) 88B:756–9. 10.1302/0301-620X.88B6.16995 16720769

[3] Ferrante MA Wilbourn AJ. Electrodiagnostic approach to the patient with suspected brachial plexopathy. Neurol Clin. (2002) 20:423–50. 10.1016/s0733-8619(01)00007-x 12152442

[4] Fox IK Mackinnon SE. Adult peripheral nerve disorders: nerve entrapment, repair, transfer, and brachial plexus disorders. Plast Reconstr Surg. (2011) 127:105e–18e. 10.1097/PRS.0b013e31820cf556 21532404 PMC3864586

[5] Limthongthang R Bachoura A Songcharoen P Osterman AL. Adult brachial plexus injury: evaluation and management. Orthop Clin North Am. (2013) 44:591–603. 10.1016/j.ocl.2013.06.011 24095074

[6] Arzillo S Gishen K Askari M. Brachial plexus injury: treatment options and outcomes. J Craniofac Surg. (2014) 25:1200–6. 10.1097/SCS.0000000000000841 25006897

[7] Caporrino FA Moreira L Moraes VY Belloti JC Gomes dos Santos JB Faloppa F. Brachial plexus injuries: diagnosis performance and reliability of everyday tools. Hand Surg. (2014) 19:7–11. 10.1142/S0218810414500026 24641734

[8] Mansukhani KA. Electrodiagnosis in traumatic brachial plexus injury. Ann Indian Acad Neurol. (2013) 16:19–25. 10.4103/0972-2327.107682 23661958 PMC3644777

[9] O'Shea K Feinberg J Wolfe SW. Imaging and electrodiagnostic work-up of acute adult brachial plexus injuries. J Hand Surg Eur Vol. (2011) 36:747–59. 10.1177/1753193411422313 21921067

[10] Levin KH Wilbourn AJ Maggiano HJ. Cervical rib and median sternotomy-related brachial plexopathies: a reassessment. Neurology. (1998) 50:1407–13. 10.1212/wnl.50.5.1407 9595996

[11] Lim SH Lee JS Kim YH Kim TW Kwon KM. Spontaneous recovery of non-operated traumatic brachial plexus injury. Eur J Trauma Emerg Surg. (2018) 44:443–49. 10.1007/s00068-017-0810-x 28656387

[12] Giuffre JL Kakar S Bishop AT Spinner RJ Shin AY. Current concepts of the treatment of adult brachial plexus injuries. J Hand Surg Am. (2010) 35:678–88. 10.1016/j.jhsa.2010.01.021 20353866

[13] Martin E Senders JT DiRisio AC Smith TR Broekman MLD. Timing of surgery in traumatic brachial plexus injury: a systematic review. J Neurosurg. (2018) 1:1–13. 10.3171/2018.1.JNS172068 29999446

[14] Martinoli C Bianchi S Santacroce E Pugliese F Graif M Derchi LE. Brachial plexus sonography: a technique for assessing the root level. AJR Am J Roentgenol. (2002) 179:699–702. 10.2214/ajr.179.3.1790699 12185049

[15] Shafighi M Gurunluoglu R Ninkovic M Mallouhi A Bodner G. Ultrasonography for depiction of brachial plexus injury. J Ultrasound Med. (2003) 22:631–4. 10.7863/jum.2003.22.6.631 12795559

[16] Graif M Martinoli C Rochkind S Blank A Trejo L Weiss J. Sonographic evaluation of brachial plexus pathology. Eur Radiol. (2004) 14:193–200. 10.1007/s00330-003-1997-2 12845468

[17] Haber HP Sinis N Haerle M Schaller HE. Sonography of brachial plexus traction injuries. AJR Am J Roentgenol. (2006) 186:1787–91. 10.2214/AJR.04.1861 16714675

[18] Gruber H Glodny B Galiano K Kamelger F Bodner G Hussl H. High-resolution ultrasound of the supraclavicular brachial plexus – can it improve therapeutic decisions in patients with plexus trauma. Eur Radiol. (2007) 17:1611–20. 10.1007/s00330-006-0464-2 17072615

[19] Chen DZ Cong R Zheng MJ Zhu T Coles G Feng H. Differential diagnosis between pre- and postganglionic adult traumatic brachial plexus lesions by ultrasonography. Ultrasound Med Biol. (2011) 37:1196–203. 10.1016/j.ultrasmedbio.2011.04.015 21645961

[20] Tagliafico A Succio G Serafini G Martinoli C. Diagnostic performance of ultrasound in patients with suspected brachial plexus lesions in adults: a multicenter retrospective study with MRI, surgical findings and clinical follow-up as reference standard. Skeletal Radiol. (2013) 42:371–6. 10.1007/s00256-012-1471-9 22707095

[21] Zheng M Zhu Y Zhou X Chen S Cong R Chen D. Diagnosis of closed injury and neoplasm of the brachial plexus by ultrasonography. J Clin Ultrasound. (2014) 42:417–22. 10.1002/jcu.22155 24677066

[22] Zhu YS Mu NN Zheng MJ Zhang YC Feng H Cong R. High-resolution ultrasonography for the diagnosis of brachial plexus root lesions. Ultrasound Med Biol. (2014) 4:1420–6. 10.1016/j.ultrasmedbio.2014.02.012 24768481

[23] Nwawka OK Casaletto E Wolfe SW Feinberg JH. Ultrasound imaging of brachial plexus trauma in gunshot injury. Muscle Nerve. (2019) 59:707–11. 10.1002/mus.26461 30847944

[24] Gu S Zhao Q Yao J Zhang L Xu L Chen W. Diagnostic ability of ultrasonography in brachial plexus root injury at different stages post-trauma. Ultrasound Med Biol. (2022) 48:1122–30. 10.1016/j.ultrasmedbio.2022.02.013 35331579

[25] Chin B Ramji M Farrokhyar F Bain JR. Efficient imaging: examining the value of ultrasound in the diagnosis of traumatic adult brachial plexus injuries, a systematic review. Neurosurgery. (2018) 83:323–32. 10.1093/neuros/nyx483 29040777

[26] Padua L Martinoli C. From square to cube: ultrasound as a natural complement of neurophysiology. Clin Neurophysiol. (2008) 119:1217–8. 10.1016/j.clinph.2008.02.005 18387335

[27] Toia F Gagliardo A D'Arpa S Gagliardo C Gagliardo G Cordova A. Preoperative evaluation of peripheral nerve injuries: What is the place for ultrasound? J Neurosurg. (2016) 125:603–14. 10.3171/2015.6.JNS151001 26799303

[28] Jakobsen RL Frederiksen AF Hellfritzsch MB Qerama E A. prospective study of high resolution ultrasound in brachial plexopathies - correlation with electrophysiological measurements. Clin Neurophysiol. (2019) 130:1144–50. 10.1016/j.clinph.2019.03.034 31096121

[29] Perlas A Chan VW Simons M. Brachial plexus examination and localization using ultrasound and electrical stimulation: a volunteer study. Anesthesiology. (2003) 99:429–35. 10.1097/00000542-200308000-00025 12883416

[30] Hsu PC Chang KV Mezian K Nanka O Wu WT Yang YC. Sonographic pearls for imaging the Brachial Plexus and its pathologies. Diagnostics (Basel). (2020) 20:324. 10.3390/diagnostics10050324 32443708 PMC7277999

[31] Ricci V Ricci C Cocco G Gervasoni F Donati D Farì G. Histopathology and high-resolution ultrasound imaging for peripheral nerve (injuries). J Neurol. (2022) 269:3663–75. 10.1007/s00415-022-10988-1 35091803

[32] Sinha S Pemmaiah D Midha R. Management of brachial plexus injuries in adults: Clinical evaluation and diagnosis. Neurol India. (2015) 63:918–25. 10.4103/0028-3886.170114 26588627

[33] Singer AD Meals C Kesner V Boulis N Gonzalez FM Umpierrez M. The Multidisciplinary approach to the diagnosis and management of nonobstetric traumatic brachial plexus injuries. AJR Am J Roentgenol. (2018) 211:1319–31. 10.2214/AJR.18.19887 30247979

[34] Krawczuk AW Huber J. Standard neurophysiological studies and motor evoked potentials in evaluation of traumatic brachial plexus injuries – a brief review of the literature. Neurol Neurochir Pol. (2018) 52:549–54. 10.1016/j.pjnns.2018.05.004 29803407

[35] Griffith JF. Ultrasound of the brachial plexus. Semin Musculoskelet Radiol. (2018) 22:323–33. 10.1055/s-0038-1645862 29791960

[36] Padua L Pasquale AD Liotta G Granata G Pazzaglia C Erra C. Ultrasound as a useful tool in the diagnosis and management of traumatic nerve lesions. Clin Neurophysiol. (2013) 124:1236–43. 10.1016/j.clinph.2012.10.024 23380690

[37] Jacobson JA Middleton WD Allison SJ Dahiya N Lee KS Levine BD. Ultrasonography of superficial soft-tissue masses: society of radiologists in ultrasound consensus conference statement. Radiology. (2022) 304:18–30. 10.1148/radiol.211101 35412355

[38] Kumar AH Kim J Sadeghi N Leversedge FJ Moorman CT Grant SA. The use of ultrasound imaging for brachial plexus injury assessment following operative clavicle repair. Can J Anaesth. (2018) 65:739–41. 10.1007/s12630-018-1076-4 29383656

