# Supplementary material for: Diagnostic performance of preoperative ultrasound for traumatic brachial plexus root injury: A comparison study with an electrophysiology study
Source: Front Neurol. 2023 Jan 6;13:1077830. doi: 10.3389/fneur.2022.1077830 (PMC9852902; doi:10.3389/fneur.2022.1077830)
Supplement: Supplementary file 1 [file Data_Sheet_1.PDF]

Appendix Table 1. Standardized Preoperative Ultrasound Examination Form

BP roots	Number of neural stumps	Distance between the first neural stump and transverse process (if number of neural stumps ≥1)	Distance between the first and the second neural stump (if number of neural stumps ≥2)	Continuity of epineurium	Continuity of fascicles	Cerebrospinal fluid	Neuroma-like enlargement	Other factors
	(0, 1, 2, …)	(cm)	(cm)	(Existent / Nonexistent)	(Existent / Nonexistent)	(Existent / Nonexistent)	(Existent / Nonexistent) (Size: cm × cm)	(Scar, abnormal bony structures, etc.)
C5								
C6								
C7								
C8								
T1								

Appendix Table 2. Standardized Preoperative Needle Electromyography Examination Form

Tested muscle	Left / Right	Insertional activity	Spontaneous activity				Motor unit action potential				Interference pattern
			Fibrillation	Positive sharp wave	Fasciculation	Myotonic discharge	Amplitude	Duration	Polyphasic	Recruitment	
Abductor digiti minimi											
First interossei											
Flexor carpi ulnaris											
Abductor pollicis brevis											
Flexor pollicis longus											
Flexor digitorum superficialis											
Flexor digitorum profundus											
Flexor carpi radialis											
Extensor indicis											
Extensor digitorum											
Extensor carpi radialis											
Brachioradialis											
Triceps brachii											
Biceps brachii											
Deltoid											
Clavicular part of pectoralis major											
Sternocostal part of pectoralis major											
Latissimus dorsi											
Serratus anterior											
Infraspinatus											
Trapezius											

Appendix Table 3. Standardized Preoperative Motor Nerve Conduction Examination Form

Tested nerve	From - to	Stimulate	Record	Conduction velocity (m/s)		Latency (ms)		Amplitude (mv)	
				Left	Right	Left	Right	Left	Right
Median nerve									
Ulnar nerve									
Radial nerve									
Axillary nerve	-			-	-				
Musculocutaneous nerve	-			-	-				
Suprascapular nerve	-			-	-				
Thoracodorsal nerve	-			-	-				
Medial pectoral nerve	-			-	-				
Accessory nerve	-			-	-				
Phrenic nerve	-			-	-				

Appendix Table 4. Standardized Preoperative Sensory Nerve Conduction Examination Form

Tested nerve	From - to	Stimulate	Record	Conduction velocity (m/s)		Latency (ms)		Amplitude (μv)	
				Left	Right	Left	Right	Left	Right
Median nerve									
Ulnar nerve									
Radial nerve									
Medial antebrachial cutaneous nerve	-			-	-				
Lateral antebrachial cutaneous nerve	-			-	-				

Appendix Table 5. Standardized Preoperative Somatosensory Evoked Potential Examination Form

Tested nerve	From - to	Stimulate	Record	Conduction velocity (m/s)		Latency (ms)		Amplitude (μv)	
				Left	Right	Left	Right	Left	Right
Median nerve									
Ulnar nerve									
